# Recent Advances in Plant Chemical Biology of Jasmonates

**DOI:** 10.3390/ijms21031124

**Published:** 2020-02-07

**Authors:** Minoru Ueda, Takuya Kaji, Wataru Kozaki

**Affiliations:** 1 Department of Chemistry, Graduate School of Science, Tohoku University, Sendai 980-8578, Japan; takuya.kaji.c6@tohoku.ac.jp (T.K.); wataru.kozaki.t6@dc.tohoku.ac.jp (W.K.); 2 Department of Molecular and Chemical Life Sciences, Graduate School of Life Sciences, Tohoku University, Sendai 980-8578, Japan

**Keywords:** jasmonates, COI1-JAZ, agonist, antagonist

## Abstract

Lipid-derived plant hormone jasmonates are implicated in plant growth, reproductive performance, senescence, secondary metabolite productions, and defense against both necrotrophic pathogens and feeding insects. A major jasmonate is (+)-7-*iso*-jasmonoyl-l-isoleucine (JA-Ile), which is perceived by the unique COI1-JAZ coreceptor system. Recent advances in plant chemical biology have greatly informed the bioscience of jasmonate, including the development of chemical tools such as the antagonist COR-MO; the agonist NOPh; and newly developed jasmonates, including JA-Ile-macrolactone and 12-OH-JA-Ile. This review article summarizes the current status of plant chemical biology as it pertains to jasmonates, and offers some perspectives for the future.

## 1. Introduction

Plant hormones are signal molecules present in low concentrations in plants that control all aspects of plant growth and development. Historically, plant hormones have been identified by conventional chemical isolation techniques combined with plant phenotype assays. However, postgenomic biology has enabled the genetic identification of previously unknown plant hormones, such as the carotenoid-derived hormone strigolactone, which was discovered by a combined biosynthesis/genetic analysis approach [1,2], and some peptide hormones, by genetic analyses of the Arabidopsis genome [3].

**Figure 1:**
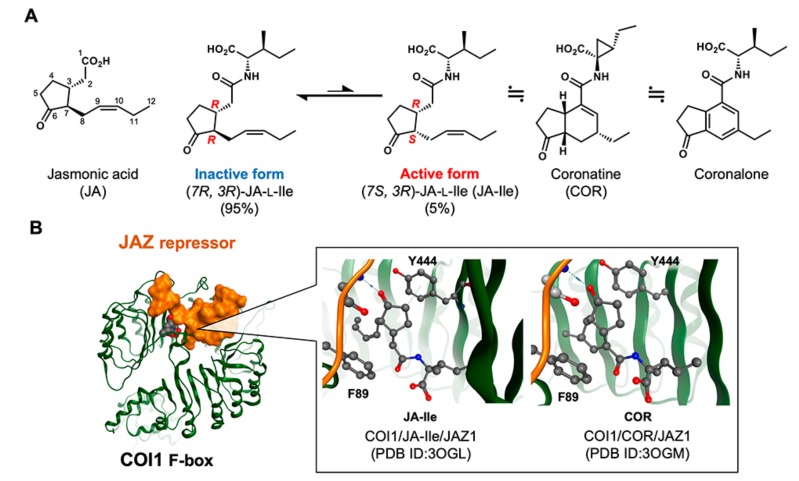
Jasmonic acid (JA) ligands and COI1-JAZ coreceptor. (**A**) Structures of JA, active/inactive form of 7-*iso*-jasmonoyl-l-isoleucine (JA-Ile), coronatine (COR), and coronalone and (**B**) comparison of the COI1 (green)/JA-Ile/JAZ1 (orange) complex (**left**, PDB:3OGL) and the COI1/COR/JAZ1 complex (**right**, PDB:3OGM) at the ligand binding pocket. ^89^Phe and ^444^Tyr sidechains of COI1 are highlighted in ball-and-stick.

Jasmonic acid (JA, Figure 1A) was first identified as a volatile component of jasmine in 1962 [4], and has been considered a plant hormone since the discovery that it causes a variety of biological responses in plants, including defense responses to attack by herbivorous insects or necrotrophic pathogens, biological responses to injury, the enhanced production of secondary metabolites, male sterility, sex-determination of plants, and growth inhibition [5,6,7,8,9,10]. The unique coreceptor system of jasmonate was disclosed by Solano’s [11], Howe’s [12], and Farmer’s groups [13], and found to entail protein–protein interactions between F-box protein CORONATINE INSENSITIVE 1 (COI1) and repressor protein JASMONATE ZIM-DOMAIN (JAZ) to form the COI1-JAZ coreceptor (Figure 1B). Subsequent degradation of JAZ repressor through a ubiquitination-guided 26S-proteasome mechanism activates the expression of the downstream gene, which is repressed by JAZ repressor. More recently, however, genetic studies concluded that (+)-7-*iso*-jasmonoyl-l-isoleucine (JA-Ile) is in fact the genuine plant hormone to which JA is merely a precursor: the JAR1 enzyme belonging to GH3 enzyme family which conjugates amino acids to diverse acyl acids is indispensable for activating the jasmonate response [14], and the jasmonate receptor COI1-JAZ has a strong affinity for JA-Ile but not JA [12]. Additionally, stereochemical studies concluded that the biosynthetic (7*S*, 3*R*)-JA-Ile (also known as (+)-7-*iso*) isomer is the genuine bioactive form that is easily epimerized to give a naturally occurring mixture of the thermodynamically stable (7*R*, 3*R*)-form and biologically active (7*S*, 3*R*)-form in a ratio of 95:5 (Figure 1A) [15].

**Figure 2:**
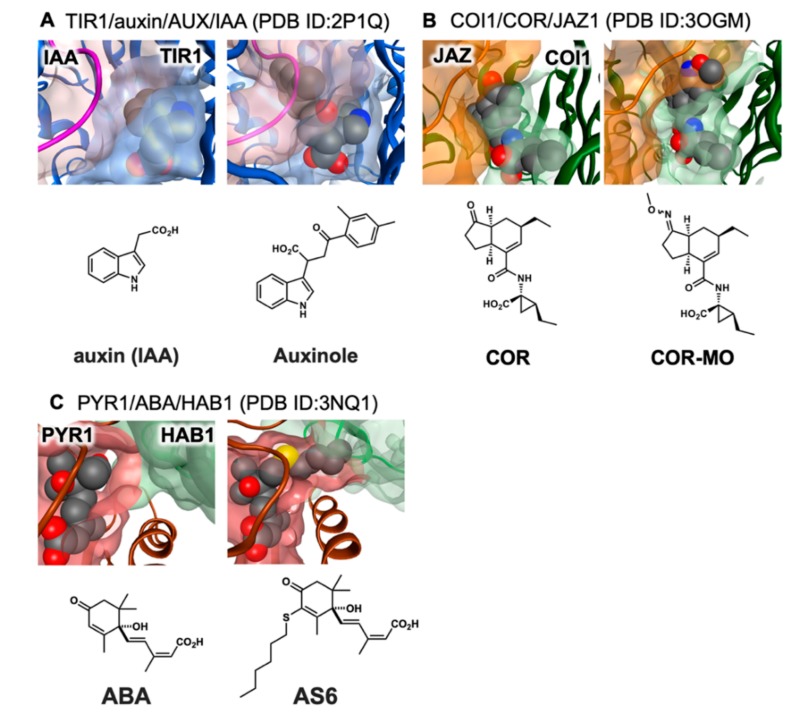
Structure-guided antagonist design. (**A**) Auxinole is superimposed onto TIR1 (blue)-AUX/IAA (purple) coreceptor (PDB:2P1Q), (**B**) COR-MO is superimposed onto COI1 (green)-JAZ1 (orange) coreceptor (PDB:3OGM), and (**C**) AS6 is superimposed for PYR1 (brown)-HAB1 (green) coreceptor (PDB: 3QN1 and 3WG8). The structures were modeled and rendered by MOE (Molecular Operating Environment, 2011.10; Chemical Computing Group Inc., Montreal, QC, Canada, 2011).

Recent advances in plant hormone biology [16], especially a vastly improved understanding of the unique coreceptor system [17], has enabled the development of chemical tools, including antagonists and agonists. Such tools are needed to probe the precise mechanistic pathways by which plants develop. The first of these was auxinole, an antagonist of auxin receptor TIR1/IAA, which paved the way for agonist/antagonist rational design (Figure 2A) [18,19]. Chemical tools have greatly contributed to our understanding and ability to control the signaling pathway of plant hormones. In this review article, we will summarize the advances in the plant chemical biology on jasmonates, and offer some perspectives for the future.

## 2. The Conventional Chemical Tools for Jasmonate Bioscience

The most important chemical tool for the study of jasmonate bioscience is the phytotoxin coronatine (COR, Figure 1A), produced by *Pseudomonas syringae *and first identified in 1977 [20]. COR is widely known as “super strong JA”, having a similar bioactivity profile to JA but an enhanced potency [21]. This enhanced potency can be understood in terms of the structure of COR, which incorporates a sTable 5-6-conjugated ring system mimicking the structure of JA-Ile, but one that cannot isomerize into (7*R*, 3*R*)-form; the analogous isomerization of (7*S*, 3*R*)-JA-Ile into the (7*R*, 3*R*)-form leads to loss of potency. In addition, the crystal structure of COI1-COR-JAZ1 complex suggests a strong hydrophobic interaction between the 5–6-conjugated ring system of COR and ^89^Phe and ^444^Tyr of COI1 (Figure 1B) [22]. COR is widely used in JA bioscience instead of JA because of the chemical stability of bioactive (7*R*, 3*R*)-form, and its most important contribution so far has been the development of the *coi1-1 *mutant, which incorporates a mutation in gene encoding the COI1 protein (a component of COI1-JAZ coreceptor) and is insensitive to COR [23]. Today, COR is considered as a structural and functional mimic of JA-Ile, and has been accessible in multigram quantities and good optical purity since the landmark work of Watson’s and Ueda’s groups [24,25,26].

One alternative to COR is coronalon, which causes JA-like responses in plants, such as volatile production and defense response against insect attack [27,28]. Coronalon is a 6-ethyl indanyl isoleucine conjugate, the synthesis of which is relatively straightforward (Figure 1A). Both coronalon and COR cause many JA responses in a variety of plants, such as Arabidopsis, lima bean, soy bean, and tobacco.

## 3. Development of Coronatine-Based Antagonist/Agonists of Jasmonate Signaling

**Figure 3:**
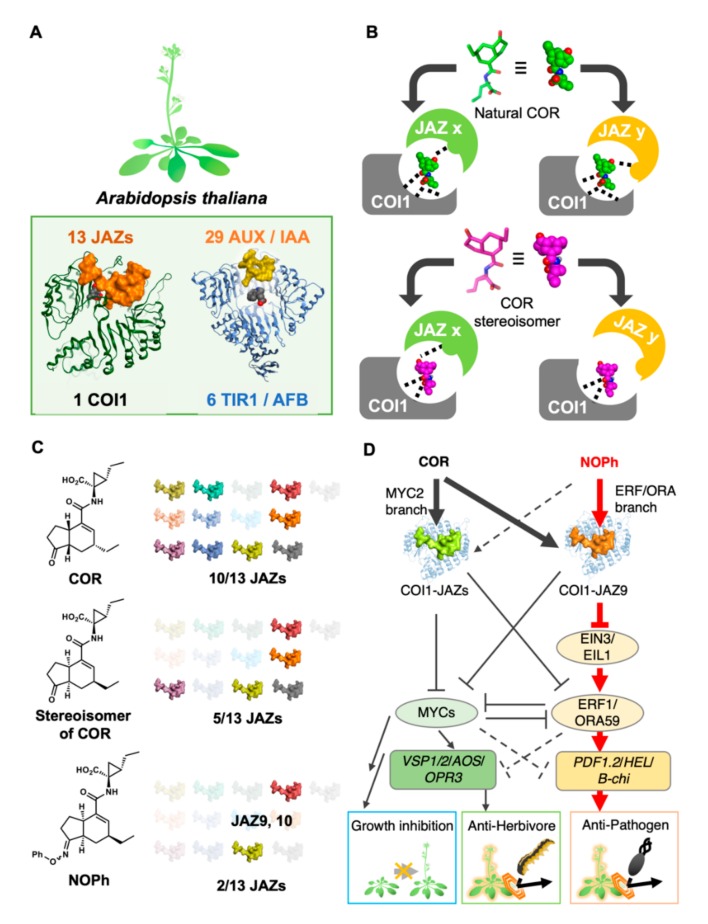
Stereochemistry-based tuning of JASMONATE ZIM-DOMAIN (JAZ) subtype selectivity. (**A**) Redundancy in plant hormone coreceptor complex; (**B**) schematic view of stereoisomer-based tuning of JAZ subtype selectivity; (**C**) observed JAZ subtype selectivity for one stereoisomer of COR and NOPh; and (**D**) overview of the mode of action of NOPh, a selective activation of ERF-ORA branch signaling pathway [49].

In general, a complete understanding of plant hormone signaling is difficult due to significant genetic redundancy of the receptor encoding genes in the plant genome [29], which precludes the exact clarification of the function of each subtype [30]. Functional redundancy among the members of such a multigene family will often hinder the genetic analysis of the contribution of individual genes using a reverse genetics strategy, because genetic knock-out of one redundant gene will be compensated for by other genes belonging to the same gene family. For example, one *COI1* gene and 13 *JAZ* genes on COI1-JAZ coreceptor of jasmonate [29], and six *TIR1/AFB* genes and 29*Aux/IAA *genes on TIR1/AFB-Aux/IAA coreceptor of auxin, are encoded in the *Arabidopsis* genome (Figure 3A). The functional *JAZ* genes are *JAZ1/2/3/4/5/6/9/10/11/12,* because *JAZ7/8/13* lack the canonical LPIARR motif in the degron necessary for the formation of COI1-JAZ coreceptor [31,32,33,34,35]. 

Chemical biology using receptor antagonists/agonists constitutes a promising solution to this problem. A general antagonist can disable all redundant signaling networks at once, and a specific agonist can trigger signaling from an otherwise redundant signaling network. However, such chemical biology studies are designed on structural information. In the context of COI1-JAZ antagonist development, Zheng’s group disclosed the crystal structure of COI1-JA-Ile/COR-JAZ1 ternary complex (Figure 1B) [22], which turned out to be very close to that of auxin receptor TIR1-IAA [36]. Interestingly, the ketone moiety of JA-Ile/COR was found to play an important role in the hydrogen bonding that causes COI1 and JAZ to interact (Figure 1B). Solano’ s group developed a rationally designed antagonist of the COI1-JAZ coreceptor system [37]—to date the only antagonist of the COI1-JAZ coreceptor—based on this important insight. The antagonist, COR-MO, is a chemically modified COR bearing a bulky methyl oxime group that protrudes from the ligand-binding pocket of COI1 and serves to impede the access of the JAZ protein, thereby inhibiting the formation of the COI1-JAZ coreceptor (Figure 2B). COR-MO effectively antagonizes the formation of COI1-JAZ complex caused by JA-Ile, and also inhibits in planta biological responses known to be caused by JA-Ile including inhibition of root elongation, anthocyanin accumulation, and the defense response against infection by necrotrophic pathogens. Previously reported antagonists of other plant hormones, such as auxinole for auxin receptor (Figure 2A) [18], and AS6 of abscisic acid (ABA) receptor (Figure 2C) [38], were also designed by inhibition of protein–protein interactions by chemical modification of plant hormone structure. The bulky alkyl chain introduced in auxinol interferes with access of Aux/IAA to the TIR1-auxinol complex, and the bulky C6-alkyl chain in AS6 interferes with the access of HAB1 to the PYR1-AS6 complex.

In contrast, progress towards the development of COI1-JAZ agonists has been much slower. One possible approach is the use of ‘biased’ agonists, which have selective affinity for the 10 genetically redundant JAZ and can be a powerful tool for the understanding of such a genetically redundant system. However, there are few successful examples of ‘biased’ plant hormone receptor agonists. Cutler’s group successfully developed the ABA receptor agonists [39] pyrabactin [40,41], quinabactin [42], cyanabactin [43], and opabactin [44], which were identified by random screening of a large-scale chemical library, and exhibited remarkable selectivity among 15 ABA receptor subtypes. The same method also resulted in the identification of SPL7, a femtomolar agonist selective for a strigolactone receptor ShHTL7 involved in the seed germination of parasitic plant *Striga hermonthica* [45].

For years, the chemical screening approach was considered the only way to develop plant hormone receptors agonists, partly because no paradigm with which to accomplish their rational design existed. However, in pioneering work, Ueda’s group succeeded in the rational design of subtype-selective agonists for the COI1-JAZ coreceptor system by using unique stereochemistry-based tuning of subtype selectivity (Figure 3B) [46]. COR as well as JA-Ile could induce protein–protein interaction (PPI) between COI1 and 10 of 13 JAZs; this multiple ligand ability of COR was attributed to the exquisite 3D structure of COR, which enabled the formation of hydrogen bond networks in all 10 possible combinations of COI1 and JAZ (Figure 3A). The slight modification of this exquisite 3D structure enabled the fine-tuning of the hydrogen bond-network (Figure 3B). The structurally modified COR could not retain a hydrogen bond-network in some of the COI1-JAZ combinations, introducing bias into its agonistic properties. Four stereochemical hybrid isomers of COR were synthesized as modified CORs, each of which could hold the same size-exclusion volume as that of original COR and could be accommodated into the small space between interreacting COI1 and JAZ. As expected, one of the four stereochemical isomers was found to have moderate selectivity (5/10) for 10 possible combinations of COI1 and JAZ (Figure 3C), and was improved using an *in silico* molecular docking strategy, resulting in NOPh that had high selectivity for 2/10 possible COI1-JAZ combinations (Figure 3C). NOPh is a phenyloxime derivative of COR stereoisomer and cause PPI between COI1 and JAZ9/10. NOPh-treated Arabidopsis showed a moderate defense response against infection by necrotrophic pathogens, without causing growth inhibition. The mode of action of NOPh was carefully examined through genetic studies, and concluded to entail selective activation of the ERF-ORA branch, one of the two major branches of jasmonate signaling pathway, through binding with COI1-JAZ9 coreceptor pair (Figure 3D). This result suggested the possible significance of chemical tools for further studies on the function of genetically redundant plant hormone receptors, and demonstrated that the transient degradation of an individual JAZ subtype might circumvent the functional compensation by other members in multigene family, which occurs in mutant plant during the development [30]. The use of a chemical tool enables the dissection of genetically redundant COI1-JAZ coreceptor function to disclose the contribution of individual subtype. Recently, Xie’s groups suggested that the affinity between COI1 and JA-Ile has been underestimated based on the observation that JA-Ile is first perceived by COI1 and the complex subsequently binds JAZs to cause a jasmonate response, and therefore the genuine receptors for JA-Ile may be COI1 [47,48]. This result may lead to new insight for the molecular design of improved agonist/antagonist of COI1-JAZ coreceptor system.

Dissection of the genetic redundancy of COI1-JAZ coreceptors has also been attempted by genetic approaches. Howe’s group elaborated the *jaz*D mutant in which 10 *JAZ* genes (*JAZ1*-7, -9, -10, and -13) are impaired [50]. Most jasmonate responses, such as upregulated defense response against necrotrophic pathogens and feeding insects, growth inhibition, poor reproductive performance, and secondary metabolite production, are observed in *jaz*D through the activation of jasmonate signaling pathway. *jaz*D can be an important background in which a* JAZ *subtype is knocked-in to examine the responsible phenotype. Solano’s group use *Marchantia polymorpha* L. instead of Arabidopsis [51,52]. No genetic redundancy is observed in the jasmonate signaling of *M. polymorpha,* because single *Mp*COI1 and *Mp*JAZ are encoded in the genome. Chemical biology using chemicals will be complementary to these two promising genetic approaches.

## 4. Protein Engineering on Ligand-Receptor Interaction in Plants

**Figure 4:**
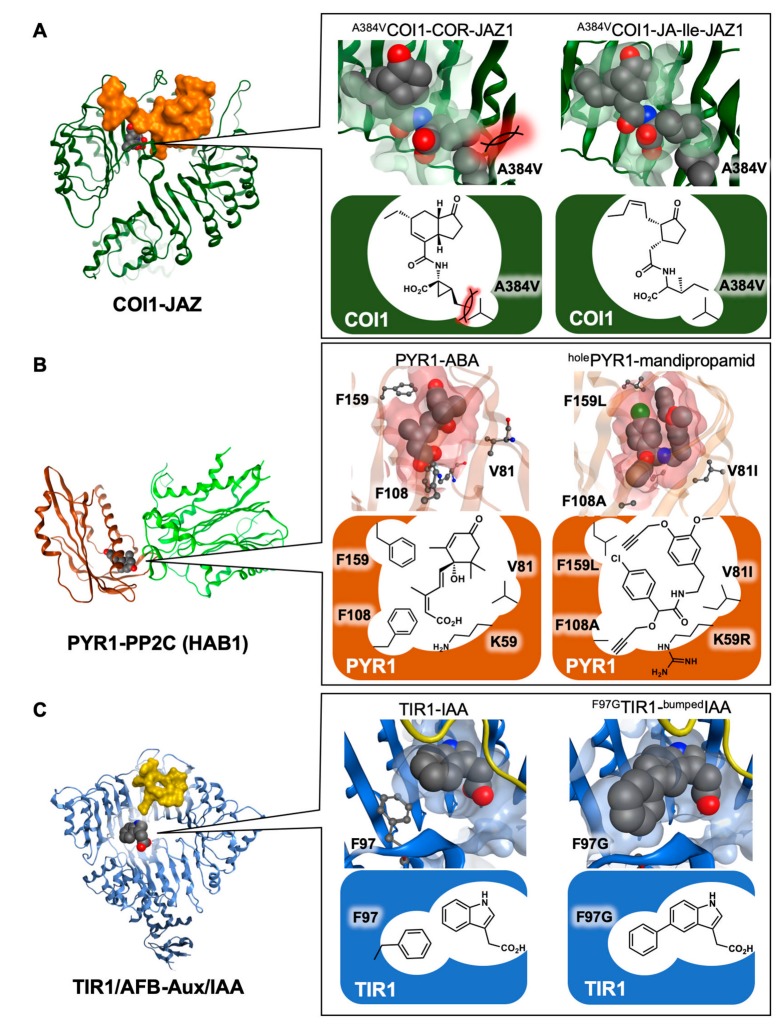
Protein engineering on ligand-receptor interaction. (**A**) Comparison of the ^A384V^COI1 (green)/COR/JAZ1 (orange) complex (**left**) and the ^A384V^COI1/JA-Ile complex (**right**) at the ligand binding pocket; (**B**) comparison of the PYR1 (brown)/ABA/HAB1 (green) complex (**left**) and the ^holed^PYR1/mandipropamid complex (**right**) at the ligand binding pocket; (**C**) comparison of the TIR1-AFB (blue)/IAA/Aux-IAA (yellow) complex (**left**) and the ^holed^TIR1/^bumped^IAA complex (**right**) at the ligand binding pocket. The structures and surfaces were modeled, docked, and rendered using MOE from (**A**) 3OGM, 3OGL; (**B**) 3QN1, 4WGD; and (**C**) 2P1Q, respectively. The ligand molecules are highlighted in sphere, and mutated residues are highlighted in either sphere or ball-and-stick, Red ball is oxygen and blue ball is nitrogen.

A particularly promising strategy for the study of plant hormone signaling is the combination of chemistry and protein engineering (Figure 4). For example, He’s group reported the breakthrough achievement of controlling the ligand selectivity of the COI1-JAZ coreceptor system [53]. This was accomplished by examination of the crystal structure of COI1-JA-Ile/COR-JAZ1, which led to the conclusion that perturbation of the shape of the ligand-binding pocket of COI1 by introduction of a point mutation (^384^Ala to ^384^ Val to give COI1^A384V^) would allow accommodation of JA-Ile, without binding to COR (Figure 4A). The flexible side chain of JA-Ile can move to avoid streric hindrance in the biding pocket. The transgenic *Arabidopsis* plant engineered to express COI1^A384V^ instead of wild-type COI1 was found to be insensitive to phytotoxin COR, and exhibited significantly increased resistance to pathogenic infection compared with wild-type plants. This result demonstrated that protein engineering techniques can affect the ligand selectivity of the plant hormone receptor. A similar strategy was also reposted by Cutler’s group [54], who modified the ligand-binding pocket of PYR1 to accommodate mandipropamid, a commercially available agrochemical. Transgenic* Arabidopsis* expressing engineered PYR1 exhibited upregulated drought tolerance (Figure 4B). In 2017, Torii and Itami’s group also applied the bump-and-hole approach [55] to auxin receptor TIR1-Aux/IAA (Figure 4C) [56]. A selective pair of bumped-auxin and holed-TIR1 was developed; among the six subtypes of TIR1/AFB, only the holed TIR1 could bind bumped-auxin to cause PPI with Aux/IAAs, although no selectivity for the possible 29 subtypes of Aux/IAA repressors was observed. Focusing on the close relationship between TIR1/AFB-Aux/IAA and COI1-JAZ receptor systems, this achievement can be also applied to jasmonate signaling to enable the selective activation of one COI1-JAZ pair among possible 10 pairs. For this purpose, a new strategy of bump-and-hole for the repressor JAZs, which correspond to Aux/IAA in auxin receptor, is necessary.

## 5. Other Chemicals Involved in the Tuned Regulation of Jasmonate Signaling

**Figure 5:**
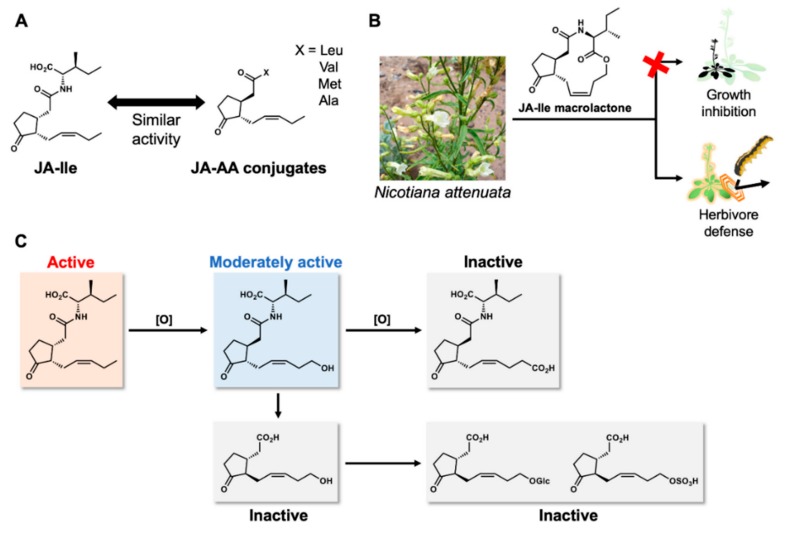
Other chemicals involved in the tuned regulation of jasmonate signaling. (**A**) JA amino acid conjugates with JA-Ile like activity; (**B**) JA-Ile macrolactone uncouples growth-defense trade-off; and (**C**) known metabolites of JA-Ile and their JA-Ile like activity, red “×” means ‘without causing growth inhibition.

The discovery of new COI1-JAZ coreceptor ligands was a landmark in jasmonate chemical biology. Some of these ligands are involved in the tuned activation of jasmonate signaling and are a possible basis for the development of chemical tools regulating the jasmonate signaling (Figure 5).

Amino acid conjugates of JA are known as conventionally-tuned jasmonates (Figure 5A). JA-Trp conjugate causes agravitropic root growth in seedlings of *A. thaliana* in a COI1-independent manner [57]. Xie’s group reported that the amino acid conjugates, JA-Leu, JA-Val, JA-Met, and JA-Ala, function as endogenous jasmonates as well as JA-Ile [58]. This result will be useful for further molecular design of COI1-JAZ agonists, the spatial limitations of the ligand-binding pocket of COI1 having been demonstrated—a finding supported by the molecular docking study by Ueda’ s group [59].

JA-Ile-macrolactone is an artificial jasmonate prepared by Boland’s group (Figure 5B) [60,61] and found to upregulate the defense response of the wild tobacco *Nicotiana attenuate* without affecting its growth. This uncoupling of growth and defense is also reported to depend on *Nicotiana COI1*. A similar phenotype was also reported by Howe’s group in *Arabidopsis* mutant impaired in quintuple *JAZs* in addition to *PhyB* [62]. The upregulation of the defense response against herbivores without growth inhibition was observed in the *jazQ phyB* mutant; the growth inhibition was found to be suppressed by jasmonate-gibberellin signaling crosstalk. One hypothesis is that JA-Ile-macrolactone affects both COI1-JAZ and the crosstalk between jasmonate and gibberellin, resulting in the uncoupling of growth and defense in *Nicotiana attenuata*.

12-Hydroxy JA-Ile (12-OH-JA-Ile) is an inactivated derivative of JA-Ile (Figure 5C) [63,64]. Hydroxylation of JA-Ile by CYP94 monooxygenases (CYP94B1/B3/C1) occurs as a late response of jasmonate signaling to lower the endogenous concentration of bioactive JA-Ile and suppress jasmonate responses [65,66,67]. Koo’s and Solano’s groups revealed that 12-OH-JA-Ile can be perceived by COI1-JAZ coreceptor with weak/moderate affinity in *Arabidopsis* and is expected to be involved in some of jasmonate responses [68,69]. Interestingly, 12-OH-JA-Ile may be selective for some JAZ subtypes and be involved in the regulation of selected jasmonate response. Considering that 12-OH-JA-Ile accumulates 10-fold higher levels than JA-Ile at a later stage and the high concentration level continues more than 8 h, the moderate and biased JAZ degradation by 12-OH-JA-Ile may fine-tune late jasmonate responses. 12-OH-JA-Ile can be further converted to 12-COOH-JA-Ile by CYP94C1 [67], 12-OH-JA by IAR3&ILL6 [70], 12-O-Glc-JA by UGT76E1 [71], and 12-HSO_4_-JA by ST2a (Figure 5C) [72]. Among them, only 12-O-Glc-JA is biologically active in a plant, causing leaf-folding movement of* Samanea saman* in COI1-independent manner [73]. However, these metabolites have no affinity with COI1-JAZ coreceptor, suggesting that 12-OH-JA-Ile may be the possible component of metabolic regulation of jasmonate response.

**Figure 6:**
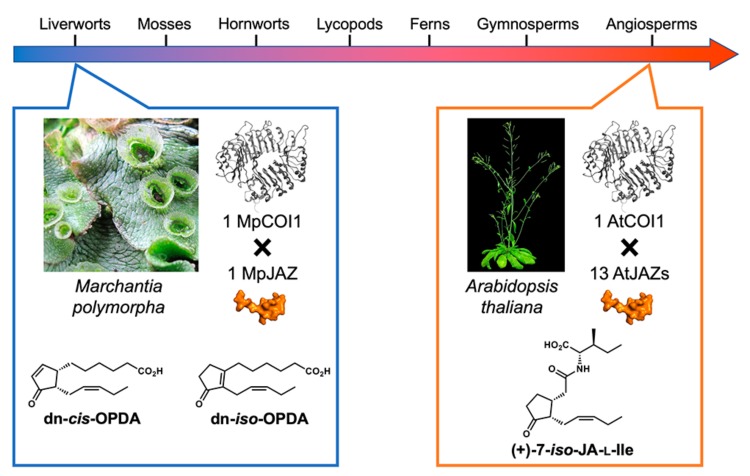
Ligand-receptor coevolution in jasmonate signaling.

One particularly intriguing research topic in plant hormone biology is the ancestral origin of hormone-receptor pair (Figure 6) [74]. The genome sequence of a bryophyte *Marchantia polymorpha* revealed the unique nature of this ancestral plant [75]. No genetic redundancy was found for jasmonate signaling components in *M. polymorpha*: one *MpCOI1* and one *MpJAZ.* Solano’s group focused on the jasmonate signaling in *M. polymorpha*, the redundant-free nature of which is convenient for genetic analyses of jasmonate signaling [51,52]. However, the unique *Mp*COI1-*Mp*JAZ coreceptor pair cannot perceive JA-Ile/COR, and the endogenous ligand is confirmed *cis*/*iso*-dinor-OPDA [76]. This is the result of unique ligand-receptor coevolution. A single point mutation in *Mp*COI1 causes this difference in ligand selectivity. Considering the high homology between *Mp*JAZ and *Arabidopsis* JAZs belonging to Group V (JAZ3/4/9) [77], *Mp*COI1-*cis*/*iso*-dinor-OPDA complex might have selective affinity with *Arabidopsis* JAZs belonging to Group V.

## 6. A Chemical Tool for Jasmonate Research from Chemical Library Screening

**Figure 7:**
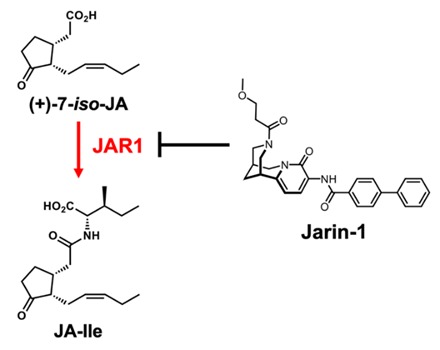
Jarin-1: the sole inhibitor of JAR1.

Jarin-1, a selective inhibitor of JAR1 enzyme, is the sole successful example of the chemical library screening on jasmonate signaling (Figure 7) [78]. JAR1 is a key enzyme of jasmonate signaling that conjugates (+)-7-*iso*-jasmonic acid to l-isoleucine to provide JA-Ile. Jarin-1 efficiently suppressed this process of JA-Ile biosynthesis and selectively impaired multiple jasmonate responses in *Arabidopsis thaliana*.

## 7. Possible Design of Chemical Tools in Non-Arabidopsis Plants

Some of the abovementioned chemical tools have been validated in non-Arabidopsis plants, but their future application is contingent upon the tuning of their chemical structures for the corresponding orthologs of *Arabidopsis *COI1-JAZs. However, no crystal structure of such orthologs exists—the COI1-JAZ1-COR/JA-Ile complex is the only exact structure reported so far. Recently, however, *in silico* homology modeling of orthologous COI1-JAZ with ligands has been reported. Boland’s group demonstrated the first application of homology modeling and docking studies on the possible structure of lima bean *Pl*COI1-*Pl*JAZ and a coronalon derivative [79]. Figueroa’s group also reported woodland strawberry *Fv*COI1-*Fv*JAZ1 and JA-Ile [80]. Further development in such studies is expected to inform the design of agonists and antagonists of orthologous COI1-JAZ coreceptor system for non-Arabidopsis plants.

## 8. Conclusions

The development of chemical tools for the regulation of plant hormone signaling is a promising field of research, and significant advances have been made in the past two decades. However, the tools developed to date are limited to the upregulation of plant defense responses against necrotrophic pathogen or insect attack, and although these are important, more versatile tools such as those that are able to improve the efficiency of plant growth are highly desirable. One research priority is a tool that is able to upregulate [79] secondary metabolite production in medicinal plants without concomitant growth inhibition [81,82], and recent studies have revealed the relationship between jasmonate signaling and transcription factors (TFs), which govern the biosynthetic genes of secondary metabolites [83,84]. Thus, chemical control of individual TF or JAZs in the upstream pathway could be an efficient strategy. Progress in the development of novel chemical tools will advance the tuning and regulation of jasmonate responses [85].
